# Cord Blood CD4^+^ T Cells Respond to Self Heat Shock Protein 60 (HSP60)

**DOI:** 10.1371/journal.pone.0024119

**Published:** 2011-09-13

**Authors:** Joost A. Aalberse, Berber Kapitein, Sytze de Roock, Mark R. Klein, Wilco de Jager, Ruurd van der Zee, Maarten O. Hoekstra, Femke van Wijk, Berent J. Prakken

**Affiliations:** 1 Department of Pediatric Immunology, Wilhelmina Children's Hospital, Utrecht, The Netherlands; 2 Department of Infectious Diseases and Immunology, Faculty of Veterinary Medicine, Utrecht University, Utrecht, The Netherlands; 3 Department of Pediatrics, Radboud University Nijmegen Medical Centre, Nijmegen, The Netherlands; New York University, United States of America

## Abstract

**Background:**

To prevent harmful autoimmunity most immune responses to self proteins are controlled by central and peripheral tolerance. T cells specific for a limited set of self-proteins such as human heat shock protein 60 (HSP60) may contribute to peripheral tolerance. It is not known whether HSP60-specific T cells are present at birth and thus may play a role in neonatal tolerance. We studied whether self-HSP60 reactive T cells are present in cord blood, and if so, what phenotype these cells have.

**Methodology/Principal Findings:**

Cord blood mononuclear cells (CBMC) of healthy, full term neonates (n = 21), were cultured with HSP60 and Tetanus Toxoid (TT) to study antigen specific proliferation, cytokine secretion and up-regulation of surface markers. The functional capacity of HSP60-induced T cells was determined with in *vitro* suppression assays. Stimulation of CBMC with HSP60 led to CD4^+^ T cell proliferation and the production of various cytokines, most notably IL-10, Interferon-gamma, and IL-6. HSP60-induced T cells expressed FOXP3 and suppressed effector T cell responses in *vitro*.

**Conclusion:**

Self-reactive HSP60 specific T cells are already present at birth. Upon stimulation with self-HSP60 these cells proliferate, produce cytokines and express FOXP3. These cells function as suppressor cells in *vitro* and thus they may be involved in the regulation of neonatal immune responses.

## Introduction

The immune system possesses a range of safekeeping mechanisms that prevent the induction of a deleterious response to self-antigens. First through negative thymic selection (central tolerance), by which T cells expressing a T cell receptor with a high affinity for self proteins are eliminated [1]. Secondly, self reactive T cells that do escape central tolerance and develop into mature T-cells are kept under control through peripheral tolerance [2]. One of the most prominent mechanisms of peripheral tolerance is mediated by regulatory T cells. Regulatory T cells (Tregs) suppress effector T cell responses in *vitro* and can down regulate an ongoing immune response in *vivo* [3]. They appear essential for the homeostasis of the immune system as deficiencies in Treg numbers and/or function lead to inflammatory diseases both in mice and in humans [4]–[6] and are already present at the start of life [7], [8]. Tregs are either directly derived from the thymus or generated in the periphery. Although the antigen specificity of Tregs is still largely unknown, it is assumed that they can act through recognition of self-antigens in the periphery [9]. This fits the premise that the immune response to certain self-antigens is important for the regulation of immune responses [10]. Cohen was the first to propose that the response to a vested group of self-antigens is important for maintaining peripheral tolerance [11]. He described the response against this limited set of self molecules in a healthy individual, formed by auto reactive T cells and antibodies as the immunological homunculus. This ‘homunculus’ of self-antigens shares various properties, among which evolutionary conservation between the self and the non-self homologue of these proteins. One of these self proteins is human heat shock protein 60 (HSP60) [12]. HSP60 is an evolutionary highly conserved cellular protein. Also, it is a stress protein and acts as a danger signal for the immune system: the expression of HSP60 is strongly up regulated following cellular stress, e.g. because of tissue damage during inflammation [13], [14]. HSP60 can activate both adaptive and innate immune responses [15], [16] which underscores that HSP60 has the potential to be a regulator in an inflammatory response. This is further supported by the observation that in both animal and human in *vitro* studies HSP60-reactive T cells have a strong immune modulatory capacity [17]–[19]. In experimental models these HSP60-specific T cells can effectively suppress in *vivo* a variety of experimental autoimmune diseases. These findings have lead to the set up of several clinical trials using HSP peptides, e.g. in rheumatoid arthritis (RA) and diabetes type I, with promising results [20]–[22].

Thus, apparently, responses to HSP60 are important for maintaining peripheral tolerance in the adult immune system. It is unknown when during live these specific T cells arise. It would seem reasonable to expect that they are primed after birth upon encounter with homologous microbial HSP in the gut [12]. However, there are indications that self-HSP60 reactivity is present at birth. As early as 1991 it was shown that cord blood cells proliferate in response to an in *vitro* challenge with the self antigen HSP60 [23], [24] and hypothesized that this reactivity is part of the normal naïve immune repertoire. A more recent study by Merbl et al [25] put these findings in a different perspective. They found that normal cord blood contains IgM and IgA autoantibodies directed against a relatively uniform set of autoantigens, such as autoantigens that are associated with autoimmune disease (such as double stranded DNA) and autoantigens related to immune regulation such as HSP60. The authors of this study suggested that this obviously benign autoimmune self reactivity, present already at birth, may have a dual function. On the one hand it may provide the basis for autoimmunity in later life, while on the other hand the “inborn” autoimmunity to regulatory self antigens such as HSP60 may actually serve to protect against autoimmune disease. Intrigued by these studies, we questioned whether CD4^+^ T cells specific for HSP60 are already detectable at birth before exposure to the microbial flora, and if so, to what type of immune response these “inborn” autoreactive CD4^+^ T cells have upon exposure to the self antigen HSP60. We found that HSP60 specific T cells are indeed present at birth. Moreover, stimulation of CBMC with HSP60 leads to CD4^+^ T cell proliferation and cytokine production, and induces T cells with an in *vitro* regulatory phenotype that are functionally suppressive.

## Results

### HSP60 induces CD4^+^T cell proliferation in cord blood

**Figure 1:**
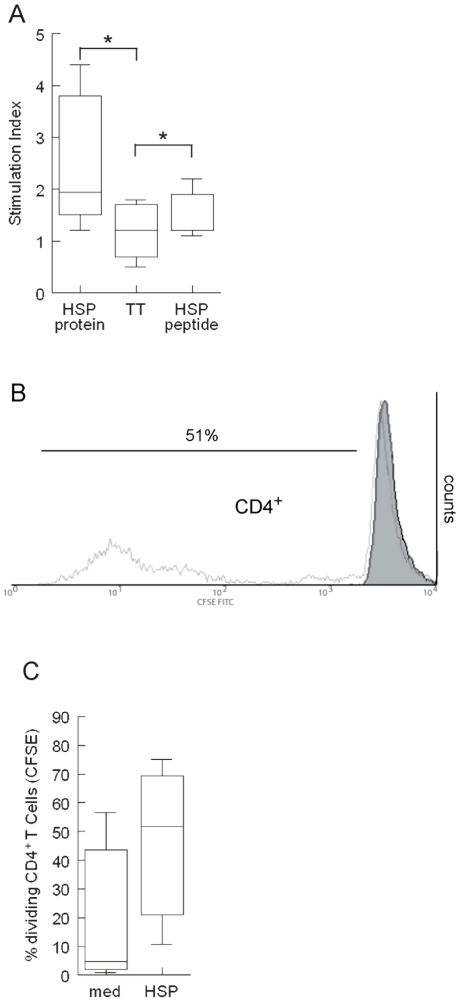
Human HSP60 (HSP) induces cell proliferation in cord blood. A) Protein-induced proliferation of cord blood mononuclear cells (CBMC). CBMC were cultured for 96 hours in the presence of Human HSP60, the full protein as well as HSP60-derived peptide and Tetanus Toxoid (TT). Box whisker plots are shown and significant differences in responses are indicated with * (p<0.05). Background (mean +/− SD): 1395 +/− 1446 CPM. Data (n = 10) were obtained from 4 independent experiments. B) Protein-induced proliferation of cord blood mononuclear cells (CBMC) cultured in the presence of CFSE. CBMC were cultured for 144 hours and afterwards stained for CD4. HSP60 proliferation is represented in lighter grey. Percentage indicates number of divided CD4^+^ T cells. Data are representative for 3 independent experiments. C) Protein-induced proliferation of cord blood mononuclear cells (CBMC) cultured in the presence of CFSE. Box whisker plots are shown. Data (n = 3) are obtained from 3 independent experiments.

To detect possible HSP60-specific T cells in cord blood, we first set out to study whether in *vitro* activation of CBMC with (human) HSP60 induces T cell proliferation. First, using thymidine incorporation we found that five of ten (50%) cord blood samples showed a clear proliferative response to HSP60 (median Stimulation Index (SI): 2.0; Inter Quartile Range (IQR): 2.5). Though we used a very pure batch of human HSP60 for these experiments with undetectable LPS levels (see methods) it is still conceivable that contamination of extremely low levels may have influenced these assays. We therefore wanted to confirm these preliminary proliferation data using two peptide epitopes from HSP60. We have shown before that these (panDR binding-) epitopes can induce T cell proliferation in a majority of patients with JIA and RA [26]. Sixty percent of the cord blood samples (three of five) responded to either one or both peptides tested (SI>1.7). These responses were also significantly different (p = 0.038) compared to the proliferative response of TT, which was used as a negative control, assuming that the fetus has not formed a prior response to TT. No significant proliferation was seen in response to tetanus toxoid (TT) compared to background. (Figure 1a).

Though this assay showed T cell proliferation to HSP60 we next wanted to confirm this with CFSE staining, which allowed us to determine which cells were dividing upon HSP60 stimulation. We found that indeed CD4^+^ T cells proliferated specifically after HSP60 stimulation in all samples (Figure 1b and 1c). Thus, HSP60 induced proliferation of cord blood derived CD4^+^ T cells.

To identify if these responses of CBMC and CD4 T cells could be the result of HSP60 acting as a ligand for TLR2 or TLR4, as previously suggested, we also performed these proliferation assays in the presence of a TLR2 and/or a TLR4 blocking antibody, or IgG isotype control (Figure S1). No differences in HSP60-induced proliferation were found in the presence of TLR blocking antibodies suggesting that the observed proliferation of CBMC in response to HSP60 is TLR2 and TLR4 independent.

### Cytokine production in cord blood stimulated cells

**Figure 2:**
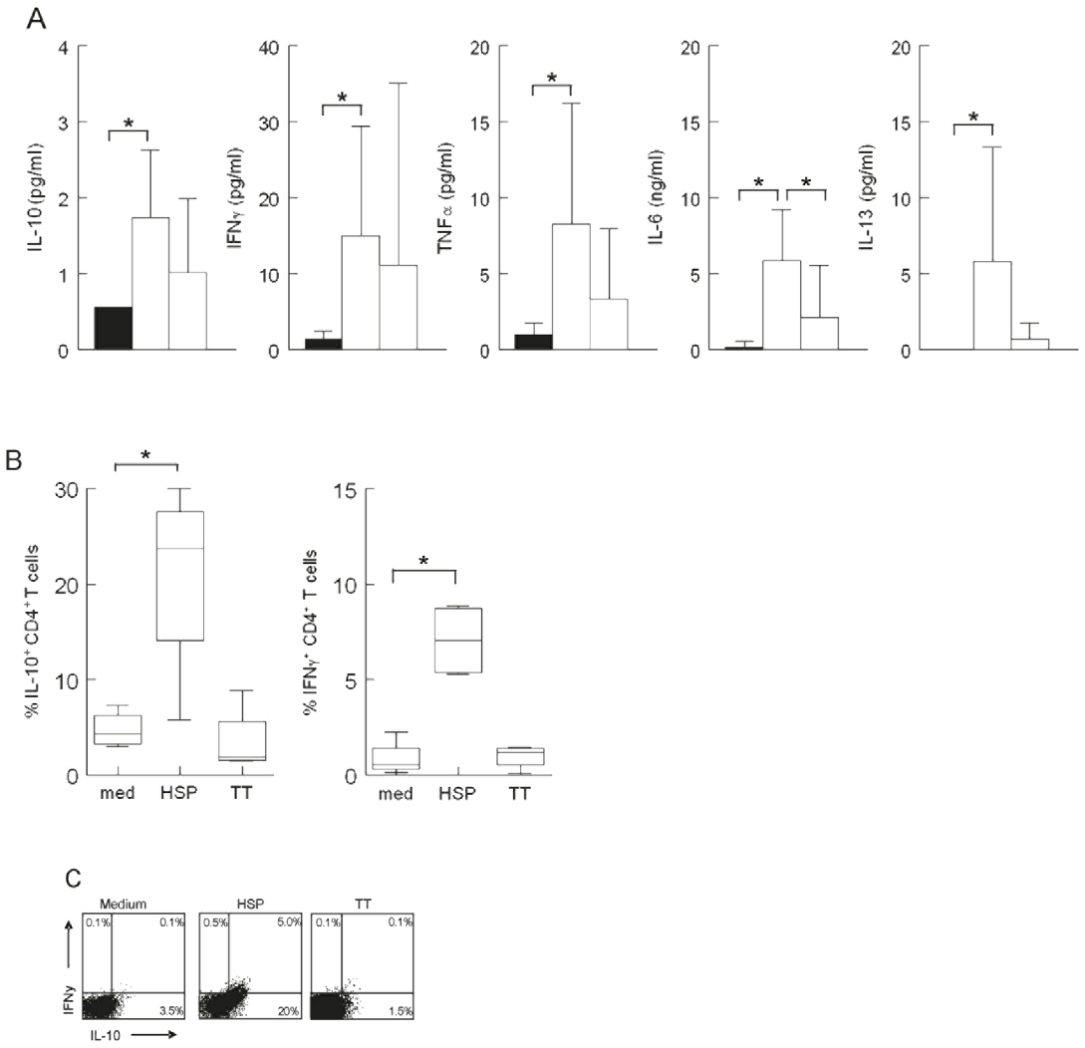
Cytokine production induced by human HSP60 (HSP) in cord blood cells. A) Cells were cultured with human HSP60 or Tetanus Toxoid (TT) for 4 days. Cytokine levels in supernatant of the cultures were measured using a multiplex immunoassay. Levels of cytokines are presented in a histogram plot. HSP stimulated cells in white, and TT stimulated cells in light grey are compared to only medium cultured cells in black. The supernatants (n = 7) were obtained in 3 independent experiments. Cytokine production was measured in one assay. B) Percentage IL-10^+^ and IFNγ^+^ CD4^+^ T cells following stimulation with human HSP60. Box whisker plots are shown and significant differences in responses are indicated with* (p<0.05). Data (n = 4) were obtained from 2 independent experiments. C) A representative FACS-analysis of two independent experiments (n = 4) showing intracellular IFNγ and IL-10 staining of CD4^+^ T cells following stimulation with human HSP60.

We next wanted to know whether stimulation with HSP60 also led to cytokine production by CBMC. Therefore, cytokine levels in culture supernatants after 96 hours of stimulation with HSP60, TT or medium alone were measured with a multiplex immune assay. We found that, compared to stimulation with medium, HSP60-induced production of IL-10 (p = 0.009), IL-5 (p = 0,013) IL-6 (p = 0.003), IL-13 (p = 0.003), TNF-alpha (TNFα) (p = 0.003) and IFN-gamma (IFNγ) (p = 0.005) were significantly increased. TT induced a significant increase of IL-6 (p = 0.045) compared to stimulation with medium (Figure 2a). To establish which cells are producing IL-10 and IFNγ, we next measured intracellular cytokine production by FACS, again after stimulation in *vitro* with medium, HSP60 and TT. We found an increased intracellular expression of mainly IL-10, and also IFNγ, by CD4^+^ T cells (Figure 2b and 2c), after stimulation with HSP60 only (and not with TT). Thus, stimulation of CBMC with HSP60 leads to proliferation and cytokine production (IL-10 and IFNγ) of CD4^+^ T cells.

### HSP60-specific induction of regulatory T cells

**Figure 3:**
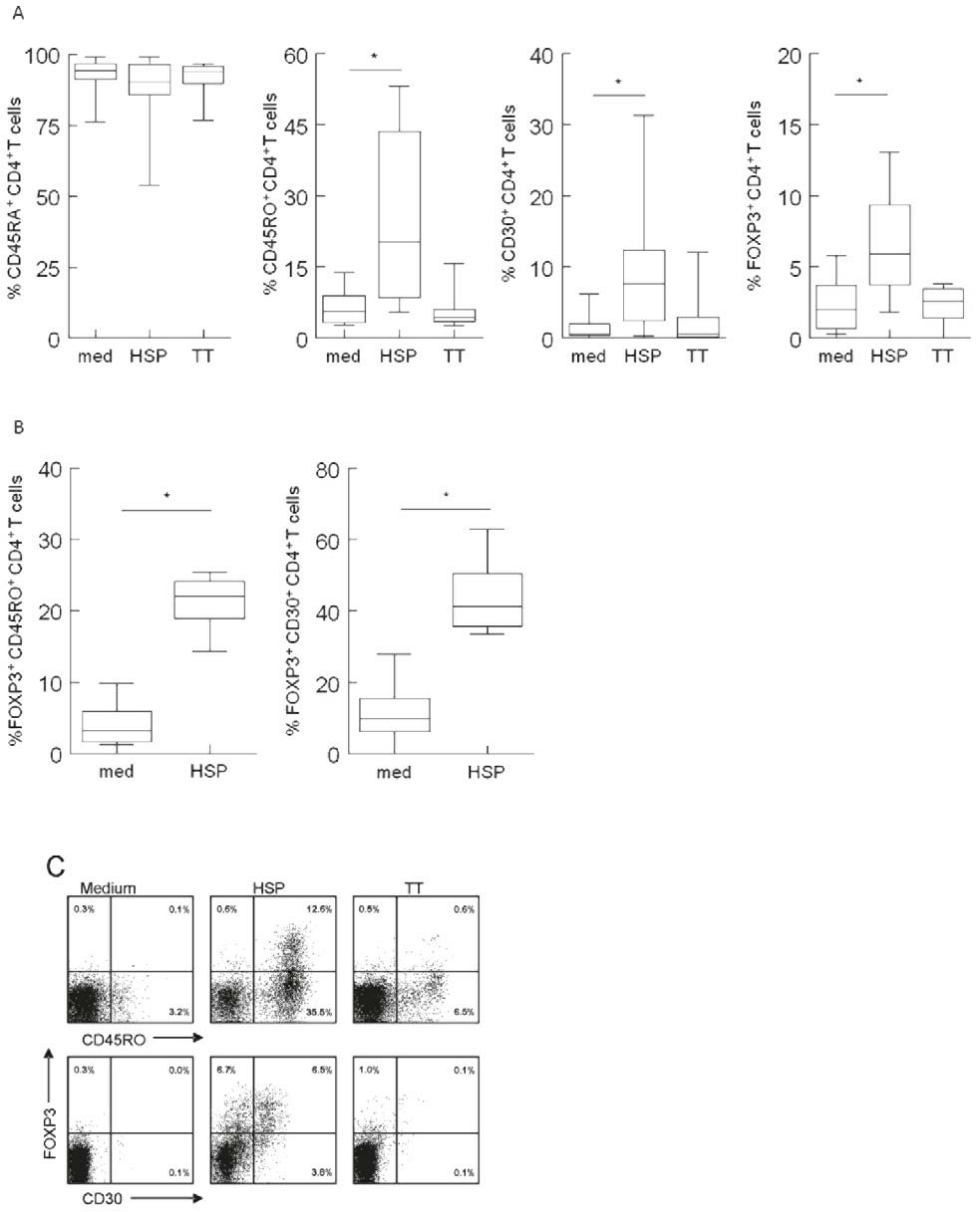
Human HSP60 (HSP) induces CD30 and the expression of Foxp3 in cord blood. CD4^+^ T cells after stimulation for 6 days. A) FACS analysis shows a significant higher level of CD4^+^CD45RA^+^, CD4^+^CD45RO^+^ and CD4^+^CD30^+^ T cells as well as CD4^+^FOXP3^+^ T cells following stimulation with HSP60. Box-Whisker plots are shown and significant differences in responses are indicated as * (p<0.05). Data (n = 7) were obtained from 3 independent experiments. B) FACS analysis of FOXP3 expression within the CD4^+^CD30^+^ and CD4^+^CD45RO^+^ populations following HSP60 stimulation. Data (n = 7) were obtained from 3 independent experiments. C) FACS analysis representing data (n = 7) from 3 independent experiments of CD4^+^ T cells expressing FOXP3 after stimulation with human HSP60, showing that most FOXP3 positive CD4 T cells also express CD45RO and half of them express CD30.

There are indications that HSP60, and/or peptides derived from HSP60 may induce or enhance the regulatory T cell population. We therefore wanted to know whether HSP60 may also play a role in T cell regulation at this crucial period at, or even before birth. We therefore stimulated cells again with TT, HSP60 or medium and next stained the cells for expression of CD4, CD30, CD45RO (memory T cells) and FOXP3. FOXP3 is a transcription factor that is characteristic for Tregs. CD30 is an activation marker that has been suggested to be a marker for Th2 cells, whereas we recently found that CD30 is upregulated on antigen-induced Tregs [27]. As can be seen in figure 3a, following stimulation with HSP60 we found a significant increase of CD4^+^CD30^+^and CD4^+^CD45RO^+^as well as an increase of CD4^+^FOXP3^+^cells, which is not seen after stimulation with control antigen TT. The FOXP3^+^ expression was found in about 50% of the CD30^+^ and 25% of the memory T cells and not in CD45RO^-^ T cells (Figure 3b and 3c).

### HSP60 induced regulatory T cells are functionally suppressive

**Figure 4:**
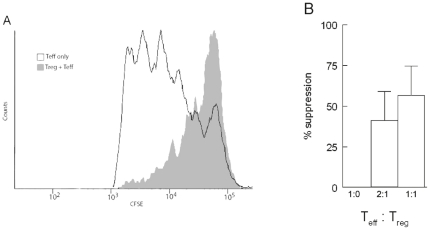
Human HSP60 (HSP)-induced CD4^+^CD25^+^ cord blood T cells suppress proliferation of T effector cells. Different amounts (0, 10.000 or 20.000) of sorted HSP60-induced regulatory T cells were cultured with 20.000 CFSE labeled effector T cells for 5 days in the presence of plate-bound anti-CD3. A) A CFSE dilution profile of effector T cells alone (open histogram) or in the presence of HSP60-induced Tregs at a ratio of 1∶1 (closed histogram). This figure represents data (n = 3) from 2 independent experiments. B) Percentages suppression measured by CFSE dilution. Data (n = 3) are obtained from 2 independent experiments.

We next questioned whether these induced Tregs were functional and thus have in *vitro* suppressive capacity. Therefore we performed CFSE dilution - suppression assays, in which we added HSP60 induced Tregs (CD4^+^CD25^+^CD127^−^ T cells) and CFSE-labeled effector cells in different ratios. The HSP60-induced Tregs were able to functionally suppress the effector T cells. Compared to the proliferating CD4^+^ T cells we saw a decreasing percentage of proliferating cells (figure 4a). After adding HSP60-induced Tregs to the effector T cells in Treg∶Teff ratios of 1∶2; and 1∶1 the percentage of suppression of proliferating CD4^+^ T cells increased from respectively 41.3 to 56.6%. This indicates that HSP60 induced CD4^+^CD25^+^CD127^−^ T cells are functional suppressor cells in *vitro*.

## Discussion

With this study we show that in cord blood HSP60 induces CD4^+^ T cells that proliferate and produce various cytokines, most notably IL-10 and IFNγ. Moreover such HSP60-induced T cells express CD30, CD45RO and FOXP3, and act as suppressor T cells in *vitro*. These findings underscore the universality and thus presumed importance of the “inborn” recognition of self proteins, like HSP60, early in life.

A healthy immune system is able to initiate as well as regulate inflammation, while maintaining tolerance for self-antigens. Immune responses to certain self proteins are hypothesized to be important for a healthy immune system [28]. These “immunological homunculus” proteins, among which stress proteins like HSP60, may serve as immune biomarkers to the body educating the immune system on the state of present inflammation. Our results would fit this hypothesis, as it suggests that an immune response to self HSP60 is part of an “inborn” mechanism present already at birth that may help to regulate inflammation. It can be speculated that human HSP60 could be regarded as a recall antigen in the fetus, as self proteins are probably continuously present in the developing fetus due to apoptosis of developing tissues. This may contribute to induction of a (tolerogenic) response to self proteins like HSP60 before birth and may explain why HSP60 in cord blood elicits such evident responses as seen in our study.

Interestingly, HSP60 has previously been identified as a potent regulator of inflammation in the adult immune system. In the end of the 80's human HSP60 was found to be recognized by the immune system during inflammation. It was then shown that HSP60-specific T cells can dampen experimental autoimmune disorders, while in humans HSP60 is recognized by healthy individuals free of autoimmune disease or infection [29]. The capacity of HSP60 to induce both innate and adaptive immunity indicates that HSP60 has a double function: responsiveness to HSP60 can be related to inflammation, but also to a healthy immune system. Next it was shown in human studies that HSP60-specific immune responses in patients with juvenile arthritis (JIA) and RA are associated with a better prognosis and milder symptoms, again suggesting a role in regulating inflammation [30]. In line with these findings we now show that HSP60-specific T cells are present at birth which underscores the hypothesis that auto-reactive T cells are a principal feature of a healthy immune system.

In addition to the finding that in cord blood HSP60 can induce FOXP3^+^ regulatory T cells, that are functionally suppressive, we have shown that HSP60 leads to the induction of IL-10 and IFNγ, suggesting the upregulation of Tr1 cells. We have also shown that HSP60 leads to the induction of Th2 cytokines (IL-5 and IL-13) confirming that an immune response in cord blood is primarily a Th2 response. Another interesting finding is the huge increase of IL-6, as well as an increase in TNFα. IL-6 has been reported as a Th2 inducer and an important anti-inflammatory cytokine, aiding in the protection of the fetus in utero [31]. Both TNFα and IL-6 however have also been shown to be upregulated by TLR agonists in cord blood. Our experiments with HSP60 peptides and our experiments in which we block TLR2 and TLR4 indicate that stimulating with HSP60 induces an antigen specific response. In conclusion, functional HSP60 specific CD4^+^ cells are already present at birth before priming of the immune cells with the microbial flora in the gut. This mechanism may be important for the maintenance of prenatal and neonatal immune tolerance.

## Materials and Methods

### Collection of cord blood

Cord blood samples (n = 21) were collected from healthy subjects at the department of gynecology and obstetrics. Inclusion criteria were an uncomplicated pregnancy, full-term infant and normal vaginal delivery. No further information about the mother, pregnancy or the delivery was obtained. Written informed consent was obtained. This study was approved by the Medical Ethics Committee, UMC Utrecht (NL16498.041.07).

### Isolation of CBMC

Cord blood mononuclear cells (CBMC) were isolated from heparinised venous blood by Ficoll (Pharmacia, Uppsala, Sweden) density gradient centrifugation. The cells were then frozen in liquid nitrogen and thawed prior the experiment. The culture was performed in RPMI-1640 supplemented with 2 mM glutamine, 100 u/ml of penicillin/streptomycin (Gibco BRL, Gaithersburg, MD) and 10% (v/v) ABpos heat-inactivated (60 min at 56° Celsius) human serum (Sanquin, Amsterdam, the Netherlands). For measurement of the proliferative activity, 2×10^5^ cells in 200 µl per well were cultured in triplicate in round bottomed 96-wells plates (Nunc, Roskilde, Denmark) for 96 hours at 37° Celsius in 5% CO2 with 100% relative humidity. Cells were cultured in the absence or presence of 10 µg/ml human HSP60, both the full protein as two peptides p2 (human HSP60 peptide 280–292) and p4 (human HSP60 peptide 242–256) [22]. The highest response to either peptide was scored. The LPS-content of HSP60 was <0.1 EU/µg protein. Tetanus-toxoid (TT; 1.5 µg/ml, RIVM, Bilthoven, the Netherlands) was used as a negative control, assuming that the fetus has not formed a prior response to TT. For the final 16 hours of culture, 1 µCi/ well [3H] thymidine (ICN Biomedicals, Amsterdam, the Netherlands) was added to each well. Cells were harvested according to standard procedures and incorporated radioactivity was measured by a liquid scintillation counter and expressed as counts per minute (cpm). The magnitude of the proliferative response is expressed as stimulation index, which is the mean cpm of cells cultured with antigen divided by the mean cpm of cells cultured without the antigen.

### TLR-blocking assay

Isolated CBMC were cultured for 96 hours in the presence or absence of 10 µg/ml human HSP60 and in the presence or absence of anti-TLR4 blocking antibody (2.5 µg/ml, clone HTA125, Serotech), anti-TLR2 blocking antibody (2.5 µg/ml, clone TL2.1, Serotech), both, or IgG2a isotype control (2.5 µg/ml, Serotech). Proliferation was measured as described above.

### CFSE assay

To characterize which cells proliferate to HSP60, CFSE-assays were performed. For this purpose 3 µM CFSE was added to 1×10^6^ CBMC for ten minutes and washed twice with 10% FCS. CBMC were resuspended in human AB 20% and stimulated with HSP60 for 6 days. Cells were then stained with anti-CD3, -CD4 and -CD14 and analyzed by FACS Calibur (Becton Dickinson Biosciences, San Jose, CA, USA), as described below.

### Multiplexed particle-based immuno assay

Cytokine levels were measured after activation of the lymphocytes in *vitro* as described above. After 96 hours the supernatants of the cell cultures were stored at −80°C. Cytokines levels of IL-4, IL-5, IL-6, IL-10, IL-12, IL-13, IFNγ and TNFα were measured with the Bio-Plex system and analysed with the Bio-Plex Manager software version 4.0 (Bio-rad Laboratories, Hercules CA, USA) which uses the Luminex xMap technology [32]. The cytokine production is presented in a color profile to visualise the different cytokines produced.

### FACS staining

CBMC were stimulated for 6 days with HSP60 and TT and then washed twice in PBS containing 2% FCS (PBS-FCS) and blocked with the appropriate normal serum (30 min at 4°C). Subsequently, the cells were incubated in 50 µl of FACS buffer containing four appropriately diluted PE-, FITC-, PerCP-, or allophycocyanin-labeled mAbs against human CD4 (clone SK3) (Becton Dickinson Biosciences), CD30 (clone BER-H8) (Becton Dickinson Biosciences), CD45RA (clone JS-83) (eBioscience, San Diego, CA, USA), CD45RO (clone 4CHL-1) (eBioscience, San Diego, CA, USA) and FOXP3 (PCH101) (eBioscience, San Diego, CA, USA). Stained mononuclear cells were diluted in FACS fluid and run on a FACS Calibur (Becton Dickinson Biosciences). CellQuest software (Becton Dickinson Biosciences) was used for analysis.

### Cytokine analysis by lymphocyte intracellular staining and flow cytometry

CBMC were stimulated as described above. During the last 4 hours of culture, Golgistop (Becton Dickinson Biosciences) was added (final concentration of 2 µM). The cells were harvested and stained for intracellular cytokine and FOXP3 analysis as described previously. For the staining, a predetermined optimal concentration of PE-conjugated anti-IL-10 (clone JES3-19F1), FITC-conjugated anti-IFNγ (clone 4S.B3) (Becton Dickinson Biosciences) and APC-conjugated FOXP3 (eBioscience, San Diego, CA, USA) was added to the cells. Cells were analysed as described above.

### Suppression assay

To examine the functionality of induced regulatory T cells, CBMC were thawed and CD25 positive cells were depleted. The CD25 negative cells were stimulated with HSP60 for 6 days. Effector T cells (thawed CBMC of the same donor) were stained with CFSE (3 µM). With a FACS sorter added different numbers of CD4^+^ CD25^+^CD127^-^ (regulatory) T cells were added to the 20.000 effector T cells, depending on availability of cells. In every experiment we included at least the Teff:Treg ratios 1∶0, 2∶1 and 1∶1. The different ratios were incubated for 120 hours in the presence of plate-bound anti-CD3 (1 µg/ml) and with the FACS diva the percentages of proliferation and suppression were measured.

### Statistical analysis

Basic descriptive statistics were used to describe the patient population. A non-parametric test (Mann Whitney U test, 2 sided) and where appropriate a parametric test (T-test, 2 sided) was applied to determine significant differences between the stimuli and the controls regarding proliferative responses, cytokine production and expression of surface markers and intracellular markers. A p-value <0.05 was considered significant (SPSS Statistical Program, version 15.0; SPSS Inc, Chicago, Ill.).

## Supporting Information

Figure S1 **Human HSP60 induced cell proliferation of CBMC, with or without anti -TLR2 and/or TLR4 blocking antibodies, or IgG isotype control.** Shown are mean stimulation index (relative to medium conditions) ± SEM. Data (n = 6) are obtained from 2 independent experiments. NS  =  non-significant.
(TIFF)
