# Supplementary figures and images for: Cord Blood CD4+ T Cells Respond to Self Heat Shock Protein 60 (HSP60)
Source: PLoS One. 2011 Sep 13;6(9):e24119. doi: 10.1371/journal.pone.0024119 (PMC3172234; doi:10.1371/journal.pone.0024119)

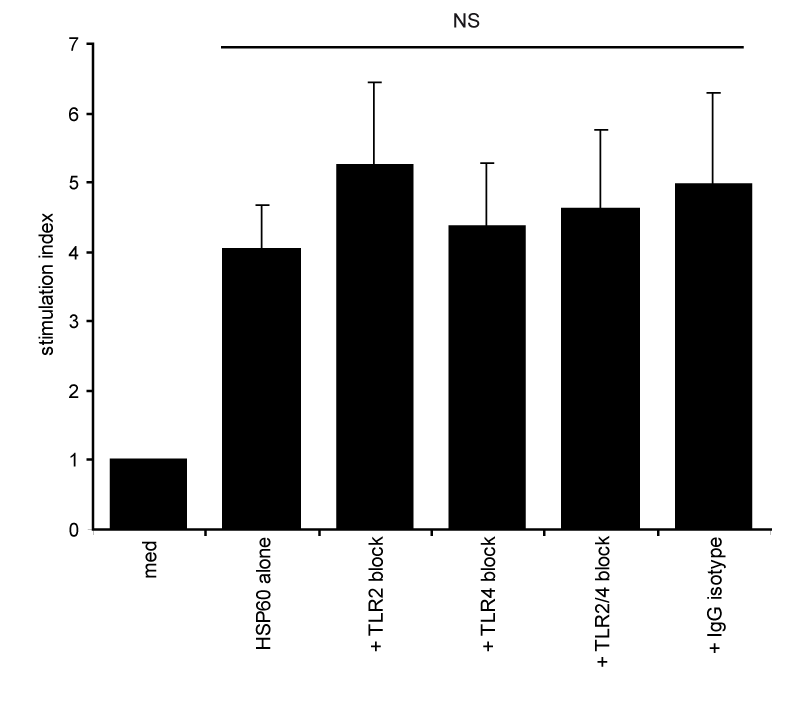

Supplement: Figure S1 — Human HSP60 induced cell proliferation of CBMC, with or without anti -TLR2 and/or TLR4 blocking antibodies, or IgG isotype control. Shown are mean stimulation index (relative to medium conditions) ± SEM. Data (n = 6) are obtained from 2 independent experiments. NS = non-significant. (TIFF) [file pone.0024119.s001.tiff]
